# Supporting translation with early health technology assessment: from definition to action

**DOI:** 10.1017/S0266462325100147

**Published:** 2025-06-02

**Authors:** Sean P. Gavan

**Affiliations:** Manchester Centre for Health Economics, Division of Population Health, Health Services Research and Primary Care, School of Health Sciences, Faculty of Biology, Medicine and Health, The University of Manchester, Manchester, UK

**Keywords:** decision-making, early health technology assessment, research and development, translation, uncertainty

## Abstract

Translating emerging health technologies towards adoption and patient benefit requires timely and effective research and development decisions. Early health technology assessment has a key role to play in supporting these decisions. A new consensus definition of early health technology assessment is a welcome contribution to help bring these activities toward wider use in the field. In parallel, the opportunities to perform early health technology assessment activities are increasing as new types of health technologies begin to enter healthcare systems globally. A greater focus on transparency of reporting, improving awareness around how early health technology assessment can impact decision-making, increased resourcing for these activities, expanding training for analysts, and encouraging collaboration between individuals across healthcare systems will be vital to strengthen the uptake of early health technology assessment from this point forward.

Navigating the roadmap to translate health technologies from their initial conceptualization to widespread adoption is challenging for innovators and product developers globally (1). The threat posed by the valley of death remains ever-present along the translational pathway to achieving patient benefit and value to healthcare systems (2;3). Advancing new health technologies through the technology readiness levels requires investment decisions into sequential research and development activities, alongside timely recognition of value drivers to guide product development. Health technology assessment (HTA) has a key role to play in supporting these decisions at all stages of the translational pathway (4). While much emphasis has been placed on HTA to inform adoption decisions for health technologies that have progressed successfully to a high level of technology readiness, there is now increasing awareness that early HTA has considerable scope to improve how decisions are made to move promising health technologies along the translational pathway (5). The definition of early HTA provided by Grutters et al. (6), with input from over 100 contributors internationally, provides an essential step for the field to emphasize the legitimacy and value of performing HTA during earlier phases of technology readiness, with a specific focus on informing subsequent development, research, and investment decisions.

Methods of analysis to facilitate HTA have been used for many years as health technologies are being developed (7). However, in most cases to date, these examples of early HTA activities have remained outside of the public domain. For example, commercial pharmaceutical manufacturers routinely undertake early analyses to inform indicative pricing decisions, internal resource allocation across product development pipelines, and priorities for evidence generation to achieve regulatory approval and reimbursement (8). In an academic setting, early analyses are also used to strengthen the case for external funding into translational evidence generation from early phase trials to later-phase pragmatic implementation research. The commercially sensitive nature of the decisions being made has been a central reason why examples within the private sector remain confidential and inaccessible outside of these organizations (9). This arrangement may be desirable if it leads to more effective and cost-effective health technologies reaching higher levels of technology readiness while protecting intellectual property. It remains unlikely that these early HTA activities informing commercial product development or pricing decisions will become available within the public domain. Yet, the case for confidentiality in the context of early HTA activities supported by public funding is less clear. These examples based on public funding may remain undisclosed for a range of reasons, including low prioritization to disseminate, insufficient staffing time to pursue dissemination, or a perception that analyses contributing to early HTA are not valuable for wider audiences. On the contrary, early HTA activities guided by public funding are likely to be highly valuable through stimulating relevant health technology innovation in the academic and commercial sectors with greater openness around target product profiles, potential market size, model-based pricing headroom analyses, valuation or design of research and development activities, and quantifying disease burden or cost of illness (6). As the field moves toward a unified definition of early HTA, a concerted effort to make the outputs from early HTA activities available more widely (when feasible) is likely to be the next step with the most leverage to help derisk the health technology translational pathway.

The source and nature of new health technologies entering the healthcare ecosystem have started to evolve in recent years. As a result, there are growing opportunities for early HTA to help improve research and development activities so that effective and cost-effective health technologies are produced within this changing ecosystem. For example, the share of new health technologies from small- to medium-sized enterprises in the digital and medical device sectors is increasing (10;11). The relatively lower barriers to entry compared with pharmaceuticals, technological advancements that reduce time to deliver a minimum viable product, and improved awareness around regulatory pathways are all likely factors contributing to growth in these sectors. Yet, these small- to medium-sized enterprises may have a lower understanding of evidence generation strategies to meet the expectations of decision-makers in healthcare systems and smaller budgets to support research and development activities. Early HTA will have a vital role in guiding these digital and medical device health technologies to higher levels of technology readiness by helping to inform research and development activities within constrained budgets and improving the chance of eventual widespread adoption rather than smaller-scale or piecemeal adoption at single provider centers. The improving feasibility of producing and delivering advanced therapy medicinal products also presents another growing opportunity for early HTA involvement (12). High-cost cell and gene therapies are now entering healthcare systems, often with limited supporting evidence at the point of adoption, which can pose challenges for decision-makers when evaluating their effectiveness, cost-effectiveness, and uncertainty (13). Embedding HTA more explicitly at earlier points within the development pathway will provide useful insights into indicative pricing, justification for first-in-human trials, and the most valuable research and development activities to help eventual adoption decisions after cell and gene therapies achieve regulatory approval.

While opportunities for early HTA to support translation are growing, the infrastructure to facilitate these analyses internationally is currently less developed than that for HTA activities informing more definitive healthcare resource allocation decisions. Improving the resourcing available for early HTA will be another essential mechanism to realize the benefits of these activities at scale. Existing in-house resourcing may not be sufficient for analysts to dedicate time toward early HTA, precluding these activities from starting and sustaining the expected prior probability of risk along the translational pathway. For health technologies developed in the public sector, bespoke funding streams for early HTA to guide research and development, proportionate to the current level of technology readiness, will lead to a step change in early HTA activity that has a clear pathway to impacting decision-making. For health technologies developed in the private sector, particularly those developed by small- to medium-sized enterprises with less experience in healthcare markets, centralized hubs to connect developers with early HTA experts will reduce search costs and barriers to undertaking these activities at earlier levels of technology readiness. In parallel, bespoke training in early HTA can be deployed at an international level to build on existing analyst capabilities and upskill in how to undertake and report these activities when informing decisions for health technologies along their development pathway.

Finally, there is now a need to engage with people outside of the immediate field of HTA specialists to emphasize the benefits of performing these activities at earlier levels of technology readiness. Achieving buy-in from clinical experts, patients, and healthcare providers will help to improve the acceptability of performing HTA activities outside of adoption decision time points. In part, this can be achieved with case studies to show examples of how early HTA has been valuable for health technologies in development. For example, case studies by Abel et al. (14) and Grutters et al. (9) explain how early HTA methods were useful for emerging health technologies in the United Kingdom and the Netherlands, respectively. In addition, communicating that having sparse data is a feature rather than a limitation of these analyses, particularly for health technologies at the initial conceptualization stage, will go far in overcoming perceived barriers to performing HTA activities along the translational pathway (15). However, the step most likely to improve perceptions of early HTA with those outside of the field will be to partner with these individuals in the research design and analysis phases explicitly. Early HTA is most effective when undertaken collaboratively with individuals across the healthcare system (16). In doing so, the spillover benefit of increasing awareness across healthcare systems into how early HTA can ultimately support translation, adoption, patient benefit, and value to healthcare systems will help to position early HTA activities as a key element to embed within research and development programs from this point forward.

The challenges and pitfalls of moving health technologies from conceptualization to adoption are known by many. Early HTA is one way to help inform decision-making along this pathway and mitigate some of the risks when moving toward higher levels of technology readiness. A new consensus definition of early HTA is a welcome contribution that will serve as a staging post to bring these activities toward wider use within the field. The growing opportunities for undertaking early HTA present a significant scope to improve research and development decision-making for health technologies within academic and commercial sectors. Greater transparency of reporting, improving awareness of how these activities can lead to impact, expanding resourcing and training, and emphasizing collaborative activities with individuals across healthcare systems will be key to moving the field from this definition of early HTA and toward action.

## References

[1] Morris ZS, Wooding S, Grant J. The answer is 17 years, what is the question: Understanding time lags in translational research. J R Soc Med. 2011;104(12):510–520. 22179294 10.1258/jrsm.2011.110180 PMC3241518

[2] Seyhan A. Lost in translation: The valley of death across preclinical and clinical divide – identification of problems and overcoming obstacles. Transl Med Commun. 2019;4(8):1–19.

[3] Fernandez-Moure JS. Lost in translation: The gap in scientific advancements and clinical application. Front Bioeng Biotechnol. 2016;4(43):1–6. 27376058 10.3389/fbioe.2016.00043 PMC4891347

[4] Llorian ER, Waliji LA, Dragojlovic N, Michaux KD, Nagase F, Lynd LD. Frameworks for health technology assessment at an early stage of product development: A review and roadmap to guide applications. Value Health. 2023;26(8):1258–1269. 36990207 10.1016/j.jval.2023.03.009

[5] Ijzerman MJ, Steuten LMG. Early assessment of medical technologies to inform product development and market access: A review of methods and applications. Appl Health Econ Health Policy. 2011;9(5):331–347. 21875163 10.2165/11593380-000000000-00000

[6] Grutters JPC, Bouttell J, Abrishami P, et al. Defining early health technology assessment: Building consensus using delphi technique. Int J Technol Assess Health Care. Forthcoming. 10.1017/S0266462325100123 PMC12178750 40452233

[7] Ijzerman MJ, Koffijberg H, Fenwick E, Krahn M. Emerging use of early health technology assessment in medical product development: A scoping review of the literature. Pharmacoeconomics. 2017;35(7):727–740. 28432642 10.1007/s40273-017-0509-1 PMC5488152

[8] Miller P. Role of pharmacoeconomic analysis in R&D decision making: When, where, how? Pharmacoeconomics. 2005;23(1):1–12. 10.2165/00019053-200523010-00001 15693724

[9] Grutters JPC, Govers T, Nijboer J, Tummers M, van der Wilt GJ, Rovers MM. Problems and promises of health technologies: The role of early health economic modeling. Int J Health Policy Manag. 2019;8(1):575–582. 31657184 10.15171/ijhpm.2019.36 PMC6819627

[10] Guo C, Ashrafian H, Ghafur S, Fontana G, Gardner C, Prime M. Challenges for the evaluation of digital health solutions-A call for innovative evidence generation approaches. NPJ Digit Med. 2020;3(10):1–14. 32904379 10.1038/s41746-020-00314-2 PMC7453198

[11] Kalinowska-Beszczyńska O, Prędkiewicz K. MedTech start-ups: A comprehensive scoping review of current research trends and future directions. PLoS One. 2024;19(8):1–24. 10.1371/journal.pone.0307959 PMC11302850 39106273

[12] Wilkins GC, Lanyi K, Inskip A, Ogunbayo OJ, Brhlikova P, Craig D. A pipeline analysis of advanced therapy medicinal products. Drug Discov Today. 2023;28(5):1–8. 10.1016/j.drudis.2023.103549 36963609

[13] Gavan SP, Wright SJ, Thistlethwaite F, Payne K. Capturing the impact of constraints on the cost-effectiveness of cell and gene therapies: A systematic review. Pharmacoeconomics. 2023;41(6):675–692. 36905571 10.1007/s40273-022-01234-7

[14] Abel L, Shinkins B, Smith A, et al. Early economic evaluation of diagnostic technologies: Experiences of the NIHR diagnostic evidence co-operatives. Med Decis Making. 2019;39(7):857–866. 31556806 10.1177/0272989X19866415

[15] Grutters JPC, Kluytmans A, van der Wilt GJ, Tummers M. Methods for early assessment of the societal value of health technologies: a scoping review and proposal for classification. Value Health. 2022;25(7):1227–1234. 35168892 10.1016/j.jval.2021.12.003

[16] Wilson M, Thavorn K, Hawrysh T, et al. Stakeholder engagement in economic evaluation: Protocol for using the nominal group technique to elicit patient, healthcare provider, and health system stakeholder input in the development of an early economic evaluation model of chimeric antigen receptor T-cell therapy. BMJ Open. 2021;11(8):e046707. 10.1136/bmjopen-2020-046707 PMC8362692 34385243

